# Let’s learn about emotions - a school based universal intervention on socio-emotional learning

**DOI:** 10.1192/j.eurpsy.2025.657

**Published:** 2025-08-26

**Authors:** T. Ståhlberg, Y. Mori, X. Zhang, T. Korpilahti-Leino, K. Mishina, T. Ristkari, S. Hinkka-Yli-Salomäki, S.-I. Ishikawa, A. Sourander

**Affiliations:** 1 Adolescent psychiatry, Turku university hospital; 2 Research centre for child psychiatry, University of Turku, Turku, Finland; 3 Faculty of Psychology, Doshisha University, Kyoto, Japan; 4 Child psychiatry, Turku university hospital, Turku, Finland

## Abstract

**Introduction:**

Socio-emotional problems are widely present in school aged children thus interfering with classroom climate and possibly leading to psychopathology. Several universal interventions exist but evidence-based and motivating interventions are still warranted.

Let’s learn about emotions was originally developed in Kyoto, Japan. It is based on cognitive behavioral therapy, positive psychology and socio-emotional learning. It is a highly structured program including 12 lessons to be taught in the classroom by teachers. It has been aimed for primary school aged children. This program was translated in Finnish and culturally adapted to be suitable for the Finnish school environment.

**Objectives:**

This study aims to produce an evidence based universal intervention that is feasible for teachers and motivating and inspirational for pupils. The aim is to increase the socio-emotional skills among pupils and teachers. Thus improvenment could be noticed within the individuals as well as in the classroom and school environment.

**Methods:**

In spring 2023 (study 1) the Finnish version of the intervention was taught to each 4th grader in the city of Hyvinkää, Finland. Data was collected from pupils, parents and teachers before and twice after the intervention with Strengths and Difficulties Questionnaire (SDQ) and questionnaires of classroom environment, school safety, bullying, feasibility and satisfaction. A single group pre-post test design was used. Qualitative interviews were carried out for parents, teachers and principals after the intervention. Effectiveness on the intervention will be studied in 2025-2026 in a larger quasi-experimental study.

**Results:**

Altogether 208 pupils and parents participated in the study 1 in Hyvinkää before and after the intervention. The results showed significant improvement in the parent-reported SDQ scores in conduct problems (0.31 points; SD 1.64, p=0-022), hyperactivity (0.37 points; SD 1.36, p=0.001), peer problems (0.19 points; SD 1.09, p=0.036) and impact of difficulties (0.98 points, SD 3.08, p<0.001). No improvement was observed in the pupil report when all pupils were icluded in the analysis. A Pearson correlation test between self-reports and parent-reports for the SDQ total score revealed significant results for both T0 (r = 0.439, p < 0.001) and T1 (r = 0.377, p < 0.001). Among the pupils with the top 20% of difficulty scores by SDQ at baseline, significant improvements were observed in the SDQ total score (1.66 points; SD = 4.94, p = 0.046), emotional problems (0.90 points; SD = 2.71, p = 0.049) and impact of difficulties (0.85 points; SD = 1.43, p = 0.005).

**Conclusions:**

Let’s learn about emotions is showing promising results and it is distinguishible from other universal interventions by its manga figures and story-like approach. Future studies are warranted in order to investigate the effectiveness.

**Disclosure of Interest:**

None Declared

